# Solvate sponge crystals of (DMF)_3_NaClO_4_: reversible pressure/temperature controlled juicing in a melt/press-castable sodium-ion conductor†

**DOI:** 10.1039/d0sc06455f

**Published:** 2021-03-01

**Authors:** Prabhat Prakash, Shylendran Ardhra, Birane Fall, Michael J. Zdilla, Stephanie L. Wunder, Arun Venkatnathan

**Affiliations:** Department of Chemistry and Centre for Energy Science, Indian Institute of Science Education and Research Pune Dr Homi Bhabha Road, Pashan Pune 411008 India arun@iiserpune.ac.in; Department of Chemistry, Temple University 1901-N 13^th^ St. Philadelphia PA 19086 USA mzdilla@temple.edu slwunder@temple.edu; Materials Science and Engineering, Indian Institute of Technology Gandhinagar Gujarat 382355 India

## Abstract

A new type of crystalline solid, termed “solvate sponge crystal”, is presented, and the chemical basis of its properties are explained for a melt- and press-castable solid sodium ion conductor. X-ray crystallography and atomistic simulations reveal details of atomic interactions and clustering in (DMF)_3_NaClO_4_ and (DMF)_2_NaClO_4_ (DMF = *N-N*′-dimethylformamide). External pressure or heating results in reversible expulsion of liquid DMF from (DMF)_3_NaClO_4_ to generate (DMF)_2_NaClO_4_. The process reverses upon the release of pressure or cooling. Simulations reveal the mechanism of crystal “juicing,” as well as melting. In particular, cation–solvent clusters form a chain of octahedrally coordinated Na^+^–DMF networks, which have perchlorate ions present in a separate sublattice space in 3 : 1 stoichiometry. Upon heating and/or pressing, the Na^+^⋯DMF chains break and the replacement of a DMF molecule with a ClO_4_^−^ anion per Na^+^ ion leads to the conversion of the 3 : 1 stoichiometry to a 2 : 1 stoichiometry. The simulations reveal the anisotropic nature of pressure induced stoichiometric conversion. The results provide molecular level understanding of a solvate sponge crystal with novel and desirable physical castability properties for device fabrication.

## Abstract 2

Stimuli-responsive “solvate-sponge”-(DMF)_3_NaClO_4_ exhibits linear chains of DMF–Na^+^ ions with ClO_4_^−^ anions in the interstitial space. At increased pressure or temperature, DMF is expelled (reversibly), resulting in a new stoichiometry-(DMF)_2_NaClO_4_.

## Graphical abstract

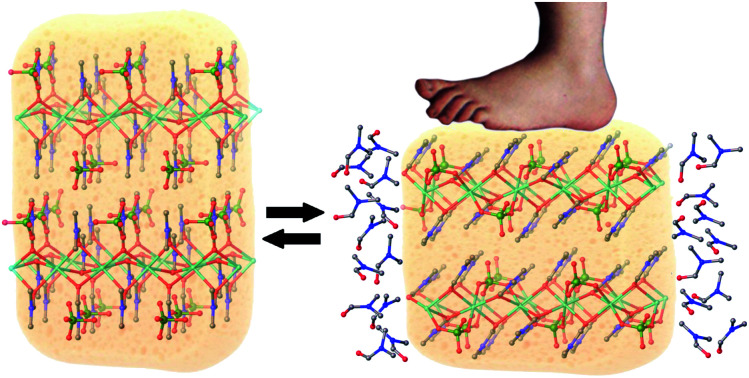

## 1. Introduction

Stimuli-response materials are an important class of substances, where mechanical actuation may be derived from the addition of energy in the form of heat, light, or force,^1–8^ combinations of heat and light,^8^ upon binding of guest species,^9^ or based on chemical and electrical stimuli.^7^ In organic heterocyclic diarylethenes, a typical *cis*–*trans* conversion *via* photoirradiation is also observed for the potential applicability as photoactuators.^8^ Stimulus responses have also been seen in self-assemblies of metal–ligand complexes, where a molecular cage facilitates the hosting of a guest molecule.^9^ In some early examples of polymer composites, cracked materials could self-heal at the crack faces, effectively reducing the brittleness of these materials.^10^ Shape retention coupled with self-healing is also seen in terephthalic acid-^11^ and terephthalamide-^12^ based crystals, where memory effects are seen as a consequence of modulation in noncovalent interactions, like intermolecular π–π stacking.^13^ Other examples of naphthalene diimides are also reported where other noncovalent interactions like hydrogen bonding, supramolecular weak interactions, and vdW forces play the major role in flexibility and shape retention.^14^ Similar to polymer composites, organic cocrystals of caffeine with other small organic molecules are also known to exhibit remarkable flexibility and bendability.^15^ Among the various examples of stimuli-response cocrystals, charge-transfer organic cocrystals have been reported.^16,17^

In this work, we consider an example from the class of salt-organic cocrystals (or salt-solvate cocrystals) of Zdilla, Wunder and co-workers: hybrid inorganic–organic systems where an inorganic salt forms soft-solid cocrystals between an organic molecule and a completely dissociative ion pair. This new class of materials exhibits Li^+^ ion or Na^+^ conduction in the solid-state.^18,19^ The immediate goal behind the synthesis of such crystals was to enable a weak interaction between alkali metal ions and their solvent matrix and to isolate and restrict the movement of anions to enhance positive-ion conduction for practical battery electrolyte applications. However, in one of the cases—where *N*,*N*-dimethylformamide cocrystalizes with sodium perchlorate as (DMF)_3_NaClO_4_,^18^—its crystal structure and morphologies have been observed to exhibit novel molecular properties, such as liquid-like grain boundaries and stimuli-responsive, reversible stoichiometric conversion to a different cocrystalline solvate species, where the application of pressure and/or temperature expels the organic solvent reversibly (the latter being the subject of this report). These cocrystals present a rare combination of coupled structural and thermomechanical behaviour and combine useful properties of other individual materials in a single example. For instance, solvent expulsion in these crystals could be comparable to a previously water respiring polymer composite.^20^ Switchable coordination (later described in results and discussion) could be similar to elastomer actuators with switchable covalent bonds,^21^ and changes in the unit cell accompanying solvent loss is related to single-crystal-to-single-crystal transformations.^22,23^

**Fig. 1:**
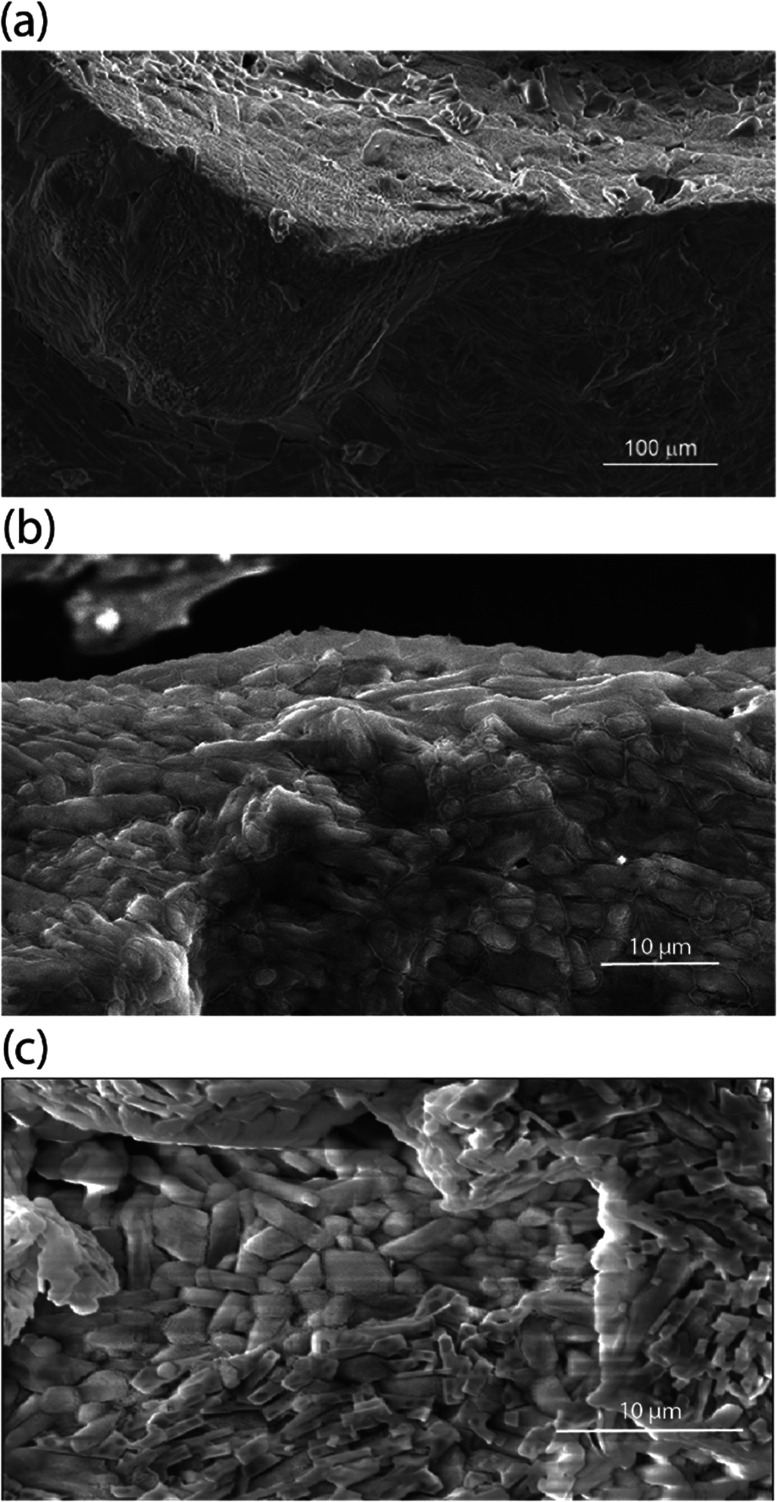
Scanning electron microgram of (a) a microcrystal of (DMF)_3_NaClO_4_ showing a contiguous array of crystallites linked by a network of nanoliquid surface layers, (b) a pressed pellet of (DMF)_3_NaClO_4_ and (c) a pressed pellet of (DMF)_2_NaClO_4_, showing a comparison of crystal orientations and morphologies. There is no liquid between crystals in pressed pellet (DMF)_2_NaClO_4_ and thus the wetting does not occur in crystalline (DMF)_2_NaClO_4_ when pressed.

In addition to the unique thermomechanical behavior, these cocrystals of DMF and NaClO_4_ exhibit a low *E*_a_ barrier for Na^+^ ion hopping (25 kJ mol^−1^, from impedance spectroscopy) and have ionic conductivity at room temperature in the range of 10^−4^ S cm^−1^ to 10^−3^ S cm^−1^.^18^ Scanning electron microscopy (SEM, Fig. 1a) suggests the presence of an inherent liquid-like region at the surface/interface of the cocrystals of these electrolytes, which also results in self-binding grains with low boundary resistance and which do not require sintering. This nanolayer results from the decreased lattice energy at the surface of the crystal, which aids in grain binding.^18,24,25^ Unlike other solid electrolytes, this grain boundary facilitates—rather than impedes—the conduction of ions across or around grains, in addition to bulk-phase ion conduction. Unlike other adiponitrile or isoquinoline based cocrystalline electrolytes,^25,26^ the DMF based electrolytes possess one-dimensional channels of closely spaced Na^+^ or Li^+^ ions, *e.g.*, (DMF)_3_NaClO_4_ (ref. 18) and DMF·LiCl.^19^ As viewed from single-crystal XRD, the cocrystals of (DMF)_3_NaClO_4_ have a one-dimensional channel of Na^+^ ions where the distance of successive Na^+^ ions is 3.23 Å, with 3 : 1 DMF : Na^+^. Such sufficiently small interionic distances can facilitate ion conduction *via* vacancy-site-induced jumps, when a cation vacancy is created during ion transport from the electrode/electrolyte or intergranular interface.

The pressure and thermal effects in the mixed inorganic-salt-in-organic-solvent matrix presented in this case are highly quantifiable as these directly manifest in a separate stoichiometry of the cocrystals. Hence, explanation of the thermomechanical behaviour in the DMF–NaClO_4_ cocrystals at macroscopic and molecular scales is a key objective of this study. Advancements in the development of electrolytes and cocrystals have been accelerated with computer simulation methods like molecular dynamics (MD) simulations, which elucidate the mechanisms of thermal decomposition and ion conduction. In previous work,^24^ thermal behavior and ion conduction in a cocrystalline electrolyte DMF·LiCl for lithium ion batteries were measured experimentally and modelled using classical MD simulations and gas phase DFT calculations. While MD simulations provided a molecular-level understanding of melting/decomposition and of a surface nanoliquid layer facilitating grain binding in pressed pellets, DFT calculations provided atomic scale explanation of ionic clusters on the surface and in the bulk phase that contribute to ionic conductivity. We report here a combined experimental and theoretical study of a new physical property of this press-castable stimuli-responsive cocrystal: (DMF)_3_NaClO_4_ (Fig. 1b), termed the “solvate sponge crystal”. Under an applied pressure, this crystalline structure “juices” to release one equivalent of liquid DMF and forms crystalline (DMF)_2_NaClO_4_ (Fig. 1c). Upon the release of pressure, the liquid DMF is reabsorbed and (DMF)_3_NaClO_4_ is re-formed in the desired pressed shape, giving a solid pellet. This behaviour is observed macroscopically (visually) as well as at the molecular level using analytical tools (XRD and thermal analysis) and computation, which all support that the introduction of pressure or temperature alters the stoichiometry of the electrolytes.

## 2. Experimental details

### 2.1 General

Single crystal and powder diffraction data were obtained on a Bruker APEX II DUO diffractometer. Mo Kα radiation was used for single-crystal structural determination, while Cu Kα radiation was used for powder diffraction studies. Thermogravimetric analysis (TGA) was carried out on a TA Instruments Hi-Res TGA 2950 at a ramp rate of 10 °C min^−1^ and under N_2_ purge gas. Differential scanning calorimetry (DSC) data were obtained on a TA Instruments Hi-Res DSC 2920 at 10 °C min^−1^ under N_2_. The samples were scanned from −110 °C to 120 °C, with the second heating scans reported. Microscope videos/photos of the pressed crystals were obtained using a Teslong NTE430 inspection camera. SEM images were obtained with a field emission gun scanning electron microscope (Quanta 450FEG SEM, FEI Co., Hillsboro, OR, USA).

### 2.2 Synthesis

*Caution*: Perchlorate-containing materials are hazardous and can cause explosions, especially at high temperature and when mixed with organic fuels. While no explosions occurred during our work, the use of explosion-proof masks, Kevlar gloves, and an explosion-proof blast shield within a fume hood is recommended when heating perchlorate–organic mixtures. See the ESI† for the standard operating procedure (SOP).

(DMF)_3_NaClO_4_ was prepared using a previously published protocol.^18^

(DMF)_2_NaClO_4_ was prepared by supercooling a solution of NaClO_4_ salt in anhydrous DMF in liquid nitrogen. 2.9 grams of NaClO_4_ (0.02 mol) and 3 ml of DMF (0.039 mol) are placed in a heavy-walled pressure flask and, using the SOP for potentially explosive mixtures, (ESI†) heated to 70 °C with stirring to increase the solubility. After all the NaClO_4_ dissolved in the solvent, the solution was rapidly frozen by immersion in liquid nitrogen, resulting in the formation of a crystalline pellet. After thawing, a few single crystals were removed from the precipitate for single-crystal structure determination. The remaining residue was removed by decanting the mother liquor, and the residue is rinsed repeatedly with diethyl ether (Et_2_O), giving (DMF)_2_NaClO_4_ (2 : 1) (Fig. S1†). The material was always contaminated by (DMF)_3_NaClO_4_ (3 : 1). The protocol gave quantitative yield of the mixture.

### 2.3 Computational details

MD simulations were performed using the Gromacs 5.0.7 (ref. 27) code using general protocols discussed below. The details of development and adaptations in force–field parameters are provided in the ESI.† A supercell consisting of 6 × 6 × 12 unit cells of (DMF)_3_NaClO_4_ (lattice parameters and coordinates obtained from Chinnam *et al.*^18^) was created in a periodic box to perform MD simulations. The 6 × 6 × 12 supercell was then converted to two different model structures: model P, where the supercell was placed in a periodic box and simulated under *NpT* ensembles, and model V, where the supercell was placed in a box with sufficient vacuum to simulate surface effects under the *NVT* ensemble. A Berendsen velocity-rescale thermostat^28^ with a time constant of 0.1 ps was used for both *NpT* and *NVT* simulations and annealing. The cocrystalline (DMF)_3_NaClO_4_ as model P was annealed in a continuous heating bath from *T* = 100 K to *T* = 500 K with a heating rate of 20 K ns^−1^. The system density and non-bonded (vdW and coulombic) components of normalized potential energy (*E*_nb_, normalized with respect to the number of pair interactions) were calculated as a function of temperature to observe the structural transformations. In the case of *NpT* simulations, Berendsen pressure coupling^29^ was used with a coupling constant of 0.1 ps. A uniform 1.2 nm cut-off was used to search neighbors and to compute vdW and coulomb forces. For all the equilibration and production runs, a timestep of 1 fs was used. In the case of semi-isotropic and anisotropic simulation trajectories to mimic the pressed pellet system, the timestep was increased to 2 fs to achieve a longer length of runtime (>50 ns).

## 3. Results and discussion

### 3.1 Thermal analysis of the stoichiometric mixture

**Fig. 2:**
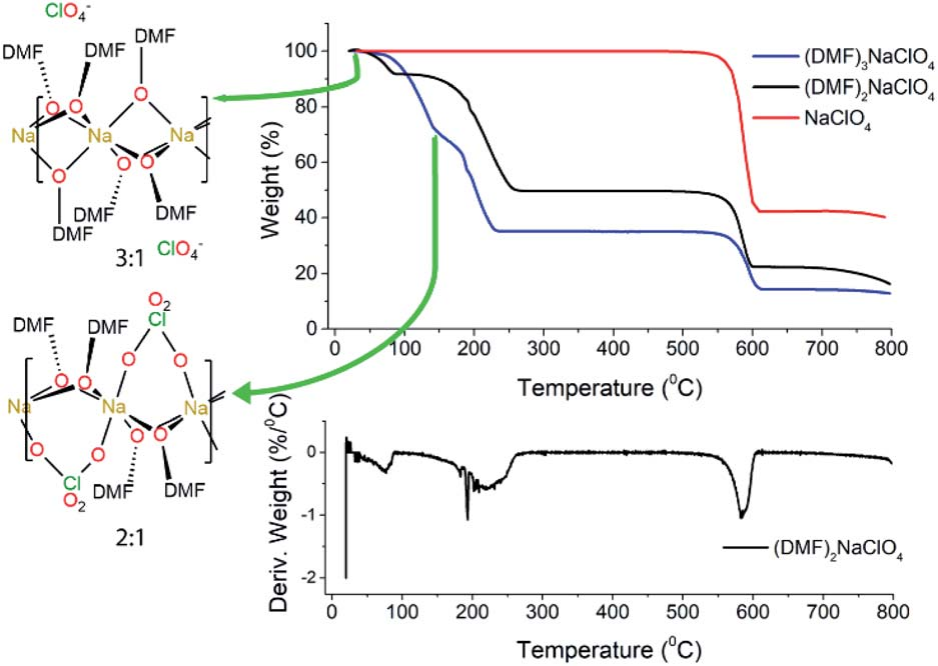
TGA and DTGA of (DMF)_*n*_NaClO_4_. TGA for 3 : 1 cocrystals is adapted from Zdilla and co-workers^18^ with its geometry shown in the left. For the (DMF)_3_NaClO_4_ solvate (blue trace), the temperatures corresponding to the 3 : 1 and 2 : 1 complexes are marked on the TGA plot.

**Fig. 3:**
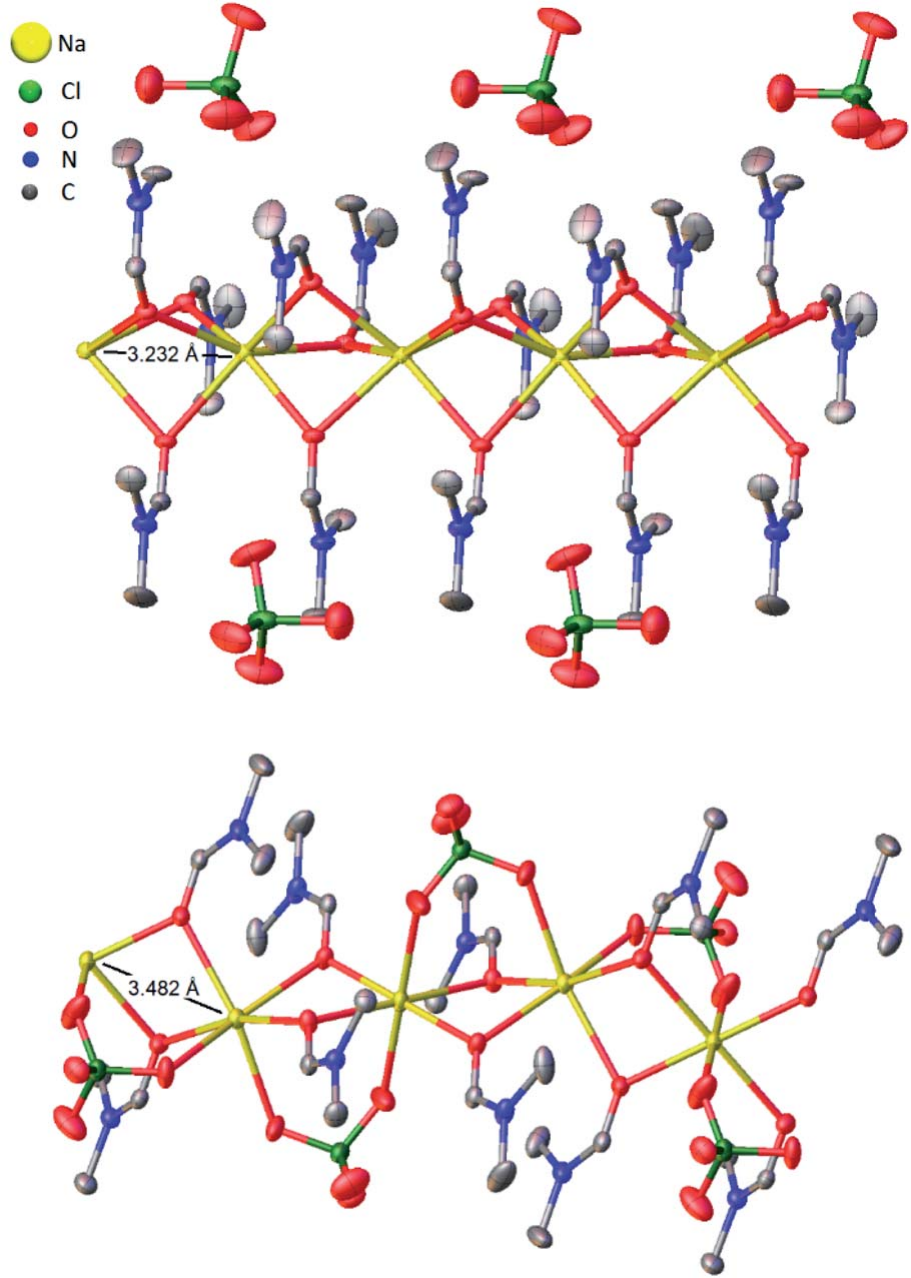
Thermal ellipsoid plots of five adjacent asymmetric units of (DMF)_3_NaClO_4_ (top)^18^ and (DMF)_2_NaClO_4_ (bottom) with Na⋯Na interatomic distances illustrated. Ellipsoids set at 50% probability level. Hydrogen atoms omitted for clarity.

Experimental studies^18^ reported that the (DMF)_3_NaClO_4_ cocrystals have a high ionic conductivity in a pressed pellet, but start melting around 55 °C. The TGA profile of the (DMF)_3_NaClO_4_ cocrystals showed a gradual decay of mass with a shoulder beginning near the melt temperature of 55 °C and ending at around 150 °C (Fig. 2). A visual inspection of the TGA of (DMF)_3_NaClO_4_ suggests the removal of the solvent (DMF) from the cocrystalline phase. The mass loss of about 25% at 150 °C is consistent with the loss of approximately one equivalent of DMF, suggesting the possibility of an isolable product with a formula (DMF)_2_NaClO_4_, a formulation that was previously reported by the group of Rao^30^ and which can be prepared in the crystalline form by heating of a stoichiometric mixture of 2 : 1 DMF : NaClO_4_ (Fig. 3).

**Scheme 1:**
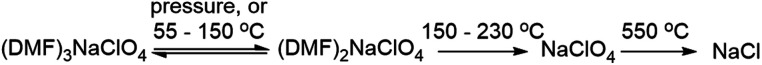
Stoichiometric changes in the cocrystals of DMF : NaClO_4_ under different conditions of temperature and pressure.

Based on the 100 K crystal structure of the 3 : 1 cocrystals, every pair of Na^+^ ions is bridged by a facial array of three DMF-κ^2^O oxygen atoms making each Na^+^ ion six-coordinate, and ClO_4_^−^ ions occupy an interstitial channel in the crystal (Fig. 3, top). In the 2 : 1 stoichiometry, one equivalent of DMF has been lost from each Na^+^ ion, and this ligand is replaced by perchlorate, which moves to a bridging position in a κ^2^O,O′ geometry, and forms a three-atom bridge across neighboring sodium ions (Fig. 3, bottom). The loss of DMF and assimilation of the perchlorate anion into the coordination sphere result in a 27% reduction of the molar volume of the crystal. The TGA result of (DMF)_2_NaClO_4_ is shown in Fig. 2. An early two-stage loss of DMF corresponds to the complete loss of DMF (54% by mass), after which the TGA shows similar results to pure NaClO_4_. At 550 °C, decomposition of NaClO_4_ ensues, leaving NaCl (an 83% total mass loss for (DMF)_3_NaClO_4_, and a 78% loss for (DMF)_2_NaClO_4_). A stoichiometric reaction scheme for these gradual mass losses is provided in Scheme 1. A list of chemical and physical properties of 3 : 1 *vs.* 2 : 1 stoichiometric cocrystals of DMF–NaClO_4_ is presented in Table S1.†

### 3.2 Pressure-induced stoichiometric conversion of cocrystals

**Fig. 4:**
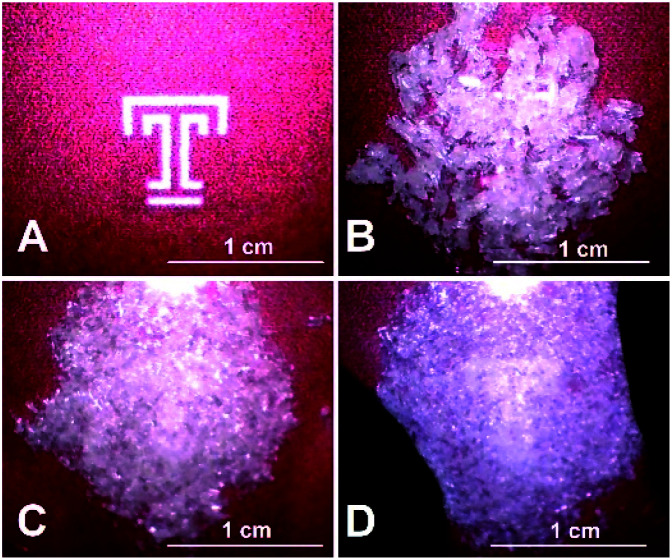
Hand pressing of solvate sponge crystals (DMF)_3_NaClO_4_ between glass slides to expel liquid DMF and generate (DMF)_2_NaClO_4_. (A) Temple T background image. (B) Fresh, dry crystals of (DMF)_3_NaClO_4_ between glass slides. (C) Initial pressing to crush the crystals, mostly obscuring the Temple T. (D) After a few minutes of pressure, the crystals become a translucent slush, with the Temple T visible beneath. See the entire process in ESI Movie 1.†

In addition to the formation of the 2 : 1 cocrystal from the 3 : 1 precursor using heat, this material may be formed from the 3 : 1 cocrystal under pressure. When a white, crystalline solid sample of (DMF)_3_NaClO_4_ is pressed between two glass slides at a pressure of about 20–30 psi (*i.e.*, “hand pressed”) for several minutes, liquid DMF is visibly expelled from the crystals (Fig. 4, ESI Movie 1†). A sample taken immediately from the edge of a mechanically pressed pellet shows the formation of the 2 : 1 DMF : NaClO_4_ crystal by PXRD analysis (see Fig. S1†). The result suggests that the 3 : 1 crystals are “juiced” under pressure to release liquid DMF and generate the reduced-volume 2 : 1 crystal.

**Fig. 5:**
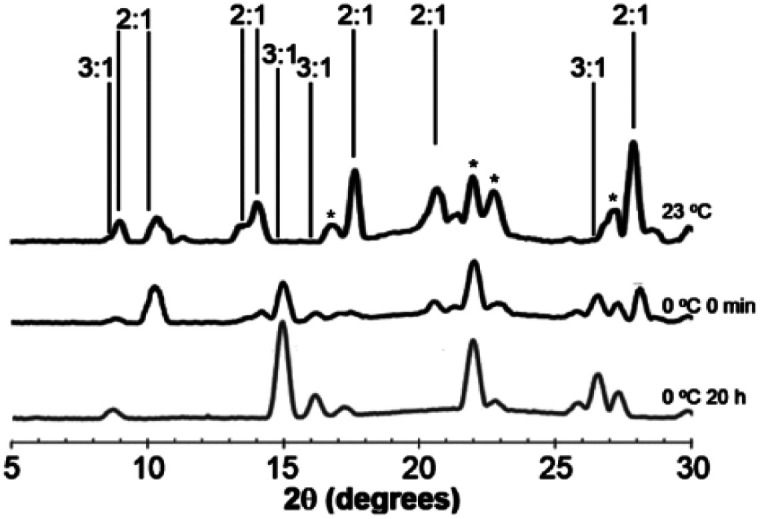
PXRD of the cooled melt of (DMF)_3_NaClO_4_ crystals. Non-overlapping PXRD lines for (DMF)_2_NaClO_4_ (2 : 1) and (DMF)_3_NaClO_4_ (3 : 1) are labelled. Overlapping peaks from both the 3 : 1 and 2 : 1 solvates are labelled with *. Top: Room temperature mixture immediately after cooling. Middle: Mixture after cooling to 0 °C, at time = 0, showing partial re-formation of the 3 : 1 mixture. Bottom: Mixture after cooling to 0 °C, at time = 20 h, showing complete reversion to 3 : 1 crystals.

Juicing of (DMF)_3_NaClO_4_ crystals also occurs thermally, and studies of the temperature dependence of these two phases of DMF–solvated NaClO_4_ suggest a dynamic equilibrium between the 3 : 1 and 2 : 1 solvates and offer clues to system thermodynamics. The 3 : 1 stoichiometry is always formed when crystals are grown from excess DMF solvent at low temperature. However, when heated past its melting temperature to 80 °C and re-cooled, the 2 : 1 solvate, (DMF)_2_NaClO_4_/DMF(l), slush forms initially, as evidenced by the PXRD pattern of this post-heated mixture after cooling to room temperature (Fig. 5, top).

Upon the release of pressure or heat from the DMF(l)/(DMF)_2_NaClO_4_ slush, the liquid DMF is reincorporated into the crystal to regenerate solid (DMF)_3_NaClO_4_ based on XRD analysis. At room temperature, over the course of 20 h, the 3 : 1 cocrystal is partially re-formed from the slush, which shows a mixture of the 2 : 1 and 3 : 1 solvates (Fig. S2†). When cooled to 0 °C, reversion of the slush to 3 : 1 is much faster and a mixture of 3 : 1 and 2 : 1 solvates is apparent immediately (Fig. 5, middle). After 20 h at 0 °C, the material completely reabsorbs all the liquid DMF, reverting to the 3 : 1 mixture (Fig. 5, bottom). At an even lower temperature of −40 °C, the reabsorption of liquid DMF from the melt is observed by XRD immediately and the 2 : 1 mixture is not detected (Fig. S2†). These results demonstrate that the 3 : 1 phase formed at low temperature is more thermally stable than the 2 : 1 mixture and that the juicing of the crystal to form the 2 : 1 mixture is endothermic. The remarkable reversibility of this solvate sponge crystal suggests that the two phases are in thermal equilibrium. Reversibility implies that the juicing process to form the DMF(l)/2 : 1 mixture is more entropically favorable, such that the phases are in equilibrium at room temperature (Δ*H* > 0, Δ*S* > 0, Δ*G* = 0). This pressure- and temperature-dependent behavior is hypothesized to be responsible for facilitating the formation of highly ionically conductive pressed or melt-cast pellets with good intergrain conductivity in the 3 : 1 (DMF)_3_NaClO_4_.^18^

### 3.3 Atomistic model for temperature induced stoichiometric conversion

**Fig. 6:**
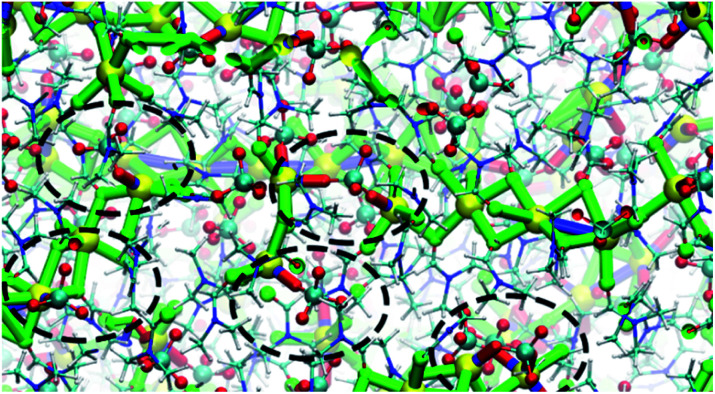
Na⋯O(ClO_4_^−^) and Na^+^···OClO_3_^−^···Na frameworks showing an abundance of Na–ClO_4_ clusters at *T* = 325 K.

MD simulations provide a molecular level description of the thermal equilibrium between the 3 : 1 and 2 : 1 cocrystals and provide a visual representation of the early stage of thermal juicing of (DMF)_3_NaClO_4_. The application of MD simulation to the 3 : 1 and 2 : 1 cocrystals provides a number of molecular insights into the behaviors of this cocrystalline material. The mass density of (DMF)_3_NaClO_4_ shows that on heating, the density decreases linearly in the temperature range *T* = 100 to *T* = 325 K, suggesting thermal expansion. However, this density decays sharply in the range 325–375 K. The graph shows a second linear decrease in density again in the range 375–500 K. This suggests that significant structural changes in the crystal interior occur in the temperature range 325–375 K. The calculated non-bonded interaction energy (*E*_nb_) with respect to temperature suggests that the Na^+^ cations, which are primarily coordinated with six DMF molecules in the cocrystals, have a higher *E*_nb_ with DMF molecules compared to ClO_4_^−^ anions, from 100–325 K. This confirms the expected role of ion–solvent interactions in the formation of 3 : 1 cocrystals. However, in the structural transformation window (*T* = 325–375 K, as seen from the mass density plot, Fig. S3†), the Na^+^ cations switch their preference of interaction from DMF to ClO_4_^−^ anions, which suggests that NaClO_4_ forms ion pairs which are either solvated by DMF ligands or phase separated from DMF. The snapshots from simulations at *T* < 325 K (Fig. S4 and ESI Movie 2†) show that the Na⋯Na (in blue) and Na⋯O(DMF) (in green) networks are more abundant in the cocrystals at low temperatures, consistent with the DMF solvation and closer Na⋯Na contacts in the experimental crystal structure (Fig. 3, top). However, at *T* = 325 K (Fig. 6), ClO_4_^−^ anions displace the DMF molecules from the coordination sphere of Na^+^ ions and coordinate in a bridged manner. As the cocrystals melt (*T* > 325 K), the Na⋯Na and Na⋯O(DMF) networks break and Na^+^···OClO_3_^−^ networks (in red) form in a significant number, suggesting the formation of NaClO_4_ ion pairs. This dynamical behavior at increased temperature is highly analogous to the experimental crystal structure of (DMF)_2_NaClO_4_ (Fig. 3, bottom), which shows lengthening of Na⋯Na vectors and replacement of a DMF ligand by ClO_4_^−^.

**Fig. 7:**
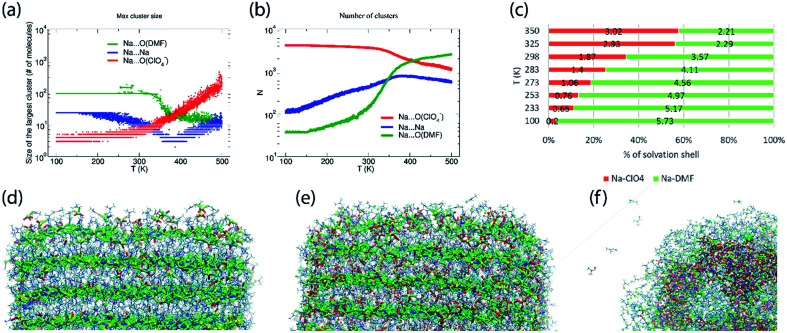
(a) Size of the largest cluster and (b) number of total clusters (counting clusters of size of one atom also) of Na⋯O(ClO_4_^−^) (≤2.2 Å), Na⋯Na (≤3.5 Å) and Na⋯O(DMF) (≤3.0 Å) during simulated heating of model P; the distribution of the number of clusters with respect to their size is provided in Fig. S6,† (c) calculated coordination number of Na^+^ ions by oxygen ligand atoms of DMF and ClO_4_^−^ anions in the cocrystalline (DMF)_3_NaClO_4_ model P at constant temperatures, simulated under the *NpT* ensemble; snapshots of the supercell of (DMF)_3_NaClO_4_ simulated as model V at (d) 100 K, (e) 300 K, and (f) 400 K. Color scheme: spheres (atoms): yellow—Na, red—O(ClO_4_), green—O(DMF), cyan—Cl(ClO_4_^−^); tubes (dynamic bonds): blue—Na⋯Na, red—Na⋯O(ClO_4_^−^), green—Na⋯O(DMF); lines—DMF. From (d) to (f), the increase in red tubes and corresponding decrease in blue and green tubes accompanies the displacement of DMF by ClO_4_^−^.

The abundance of these Na–ClO_4_ networks as a function of temperature is quantified from cluster analysis using the simulation trajectory (Fig. 7a and b). The cluster analysis shows that {Na⋯3O(DMF)}_*n*_ networks of 96 atoms (consisting of Na and O(DMF) atoms) form parallel to the *z*-axis (*c*-crystallographic direction) of the simulation box at *T* < 300 K. This network also represents the prevalence and stability of the Na^+^ ion channel in the *z*-direction. At low temperatures (*T* < 300 K), the size of the largest Na–DMF and Na–Na clusters is large (Fig. 7a) and the total number of these clusters is small (Fig. 7b). The size of the largest of Na–DMF and Na–Na clusters decreases with increasing temperature and the number of these clusters increases, which affirms the visual observation of breaking of Na–DMF and Na–Na networks at *T* > 300 K (Fig. S4†). Conversely, at *T* < 300 K, many (∼2000) small (<10, atoms at most) Na–ClO_4_ clusters exist in the system (most of which constitute a single contact), which increase in size and decrease in number as the temperature increases, suggesting the formation of large Na–ClO_4_ clusters (size >1000 atoms) at higher temperatures.

The simulated heating approach is used to calculate the interplay of non-bonded interactions during melting, which is also consistent with the loss of DMF ligands and replacement of these contacts by perchlorate bridges. To understand the distribution of clusters with respect to their size, model P was simulated under the isothermal–isobaric ensemble at constant temperatures: *T* = 100 K (20 ns), 233 K (40 ns), 273 K (40 ns), 298 K (40 ns), 325 K (40 ns), and 350 K (20 ns). The histograms of the distribution of different sized clusters at various constant temperatures (Fig. S5†) show that the Na–Na and Na–DMF clusters are predominantly abundant at low temperatures. In contrast, most of the Na–ClO_4_ clusters are monoatomic (*i.e.* only 0 or 1 interionic contact) at low temperatures, which increases to a size of 10 atoms (*i.e.* three to four ion pairs per cluster) at *T* = 325 K. The cluster analysis also suggests that Na–ClO_4_ clusters, which form after the Na⋯O(DMF) networks collapse, are small in size, indicating the solvation of small clusters of ion pairs in DMF, rather than phase separation.

To understand the nature of pair interactions in the structure of (DMF)_3_NaClO_4_, the radial distribution function (RDF) is calculated at various temperatures for model P †. The RDFs suggest that in the first solvation shell of Na^+^ cations, DMF molecules occupy the shell at a distance of 3 Å to 3.5 Å, at *T* = 100 K, whereas ClO_4_^−^ anions do not interact with the Na^+^ primary solvation shell and only interact at a distance > 5 Å. However, the Na⋯O(DMF) coordination number decreases as the temperature increases from 233 K to 298 K (Fig. 7c). In the coordination sphere of the Na^+^ cation, ∼2 O(DMF) is replaced by ∼2 O(ClO_4_^−^) contacts at a distance of 2 Å, at 298 K. This implies that (DMF)_2_NaClO_4_ could also form from (DMF)_3_NaClO_4_, provided that the residual DMF is removed from the system. The simulations do not model the exclusive formation of crystalline (DMF)_2_NaClO_4_ within these short timescales but show that under dynamical conditions, the cluster analysis and calculated coordination numbers indicate that the 3 : 1 complex is most stable at low temperature and that DMF replacement by perchlorate is increasingly favorable at higher temperatures (endothermic). As the crystals melt, at *T* = 325 K, 350 K, coordination of ClO_4_^−^ supersedes the coordination of DMF around Na^+^ cations. Overall, the *T*_m,sim_ = 325 K (= 52 °C) predicted from annealing simulations, cluster analysis and RDFs matches closely with the previously reported^18^ experimental *T*_m,exp_ = 55 °C (Fig. S7†).

While model P mimics the interior behavior of the cocrystals, the nature of the surface of (DMF)_3_NaClO_4_ was modeled using model V. Model V was constructed by placing the 6 × 6 × 12 supercell into a larger box of 15 × 15 × 18 nm^3^ with sufficient vacuum present at the either side of the supercell to avoid any significant interactions with its periodic image. From the simulated annealing of model V from *T* = 100 K to 500 K, with a heating rate of 20 K ns^−1^, a visual inspection was sufficient to extract these valuable insights: (1) at *T* = 100 K, the surface of cocrystals is fluid, with the presence of Na–ClO_4_ clusters and free DMF molecules on the surface, which are rare in the bulk (Fig. 7d); (2) at room temperature, the surface becomes even more abundant with Na–ClO_4_ clusters and free DMF, and new Na–ClO_4_ networks also become significant in the bulk (Fig. 7e); (3) at *T* = 400 K, the DMF molecules aggregate on the surface and some of them evaporate (analogous to thermal juicing and the observed mass loss during TGA) and more and larger Na^+^···ClO_4_^−^ clusters form, resulting from DMF loss (Fig. 7f). This behavior mimics well the thermal juicing and ultimate decomposition behavior of (DMF)_3_NaClO_4_ observed experimentally.

### 3.4 Atomistic model for pressure-induced stoichiometric transformation

**Fig. 8:**
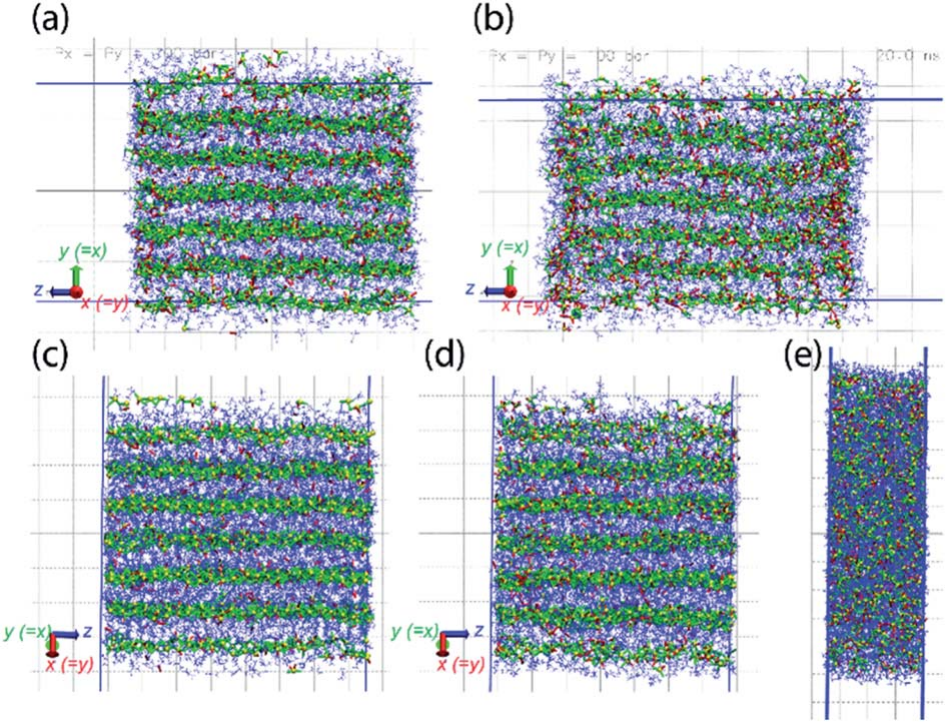
Model V simulated under semiisotropic coupling, at *P*_*x*_ = *P*_*y*_ = 100 bar, *P*_*z*_ = 1 bar, at (a) *t* = 0, (b) *t* = 20 ns; at *P*_*x*_ = *P*_*y*_ = 1 bar, *P*_*z*_ = 100 bar, at (c) *t* = 0, (d) *t* = 100 ps, (e) *t* = 1 ns. The compressibility of cocrystals is higher in the *z*-direction (bottom) compared to that in the *xy*-directions (top) as it only takes a few picoseconds to compress the crystals along *z* (note that due to hexagonal crystallographic symmetry, the *x* and *y* directions are equivalent). The simulation trajectory of compression events is provided as ESI Movie 3.†

MD simulations also demonstrate the pressure-induced juicing of the 3 : 1 crystals. Model V was simulated to mimic pressure effects under *NpT* conditions with semiisotropic pressure coupling (ESI Movie 3†). The pressing of cocrystals in the simulation box was performed using two models of pressure coupling: (1) rod-like slab: high pressure coupling from the *x* and *y* directions (*P*_*x*_ = *P*_*y*_ = 100 bar, *P*_*z*_ = 1 bar) keeping the *z* dimension sufficiently large and uncompressible and (2) rectangular plate slab: high pressure coupling from the *z* direction (*P*_*z*_ = 100 bar) keeping *xy* dimensions sufficiently large and uncompressible (*P*_*x*_ = *P*_*y*_ = 1 bar). The simulations show that the pressability of crystals depends on the direction of pressure. High pressure from *xy* directions deforms the crystals slowly as the structural integrity sustains for a longer simulation time (>10 ns) (Fig. 8a and b). Although this pressure from the *x* and *y* directions does not deform crystals as significantly, it forces ClO_4_^−^ ions to occupy the Na^+^ ion solvation sphere and forces DMF out the side of the slab, resulting in a thin surface layer of DMF (Fig. 8b). In contrast, high pressure in the *z* direction squeezes the crystals within a nanosecond, leading to the deformed structure (Fig. 8c–e). Pressure from the *z*-direction acts to break two to three (out of six) Na–DMF contacts and facilitates two to three new Na–ClO_4_ contacts. Although the classical simulations do not illustrate the full 3 : 1 to 2 : 1 stoichiometric conversion on these timescales, rapid compression observed in the *z* direction suggests that the Na–(O)DMF–Na chains are highly compressible. On compression, these chains expel one DMF molecule and coordinate with two oxygen atoms of the perchlorate anion, a behavior analogous to the observed crystal structure (Fig. 3). High pressure from the *xy* directions only presses these chains toward their neighbors, and hence, the compressibility of crystals in this direction is not as significant, although some expulsion of DMF from the Na–DMF chain is observed. Thus, soft Na–DMF interactions can account for the solvate sponge nature of DMF–NaClO_4_ crystals.

**Fig. 9:**
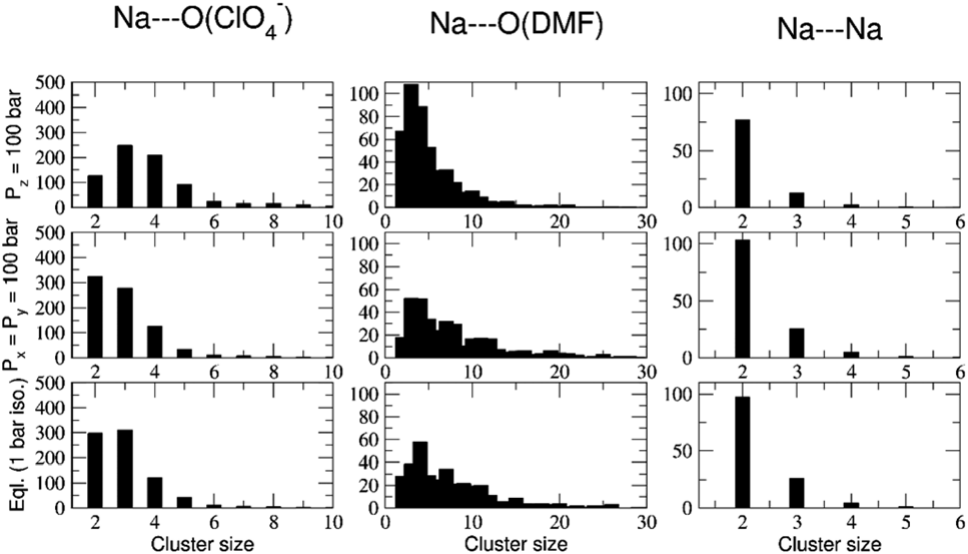
Number (*y*-axis) *vs.* size (*x*-axis) histogram showing the distribution of various sized (in columns) Na⋯O(DMF) (≤3.0 Å), Na⋯OClO_3_ (≤2.2 Å), and Na⋯Na (≤3.5 Å) clusters from an 18–20 ns time window of a 20 ns trajectory simulated under *NpT* conditions, at *T* = 298 K. Pressure conditions are arranged in rows.

A quantitative demonstration of pressure effects (similar to thermal effects) on cocrystals is shown from cluster size and number analysis during compression in Fig. S8 and S9.† A cluster histogram is shown in Fig. 9 which exhibits the effect of pressure anisotropicity in a time-averaged manner. The average size of Na–DMF clusters is ∼40 atoms (counting only Na and O atoms) at 1 bar pressure. On applying a high pressure in the *x* and *y* directions (*P*_*x*_ = *P*_*y*_ = 100 bar, *P*_*z*_ = 1 bar), the average cluster size decreases to 10 atoms, suggesting low-to-moderate chain-breaking in these crystallographic directions (Fig. S8a†). On applying a high pressure in the *z* direction (*P*_*x*_ = *P*_*y*_ = 1 bar, *P*_*z*_ = 100 bar), the average cluster size is ∼6 atoms, suggesting small fragments forming with significant Na–O(DMF) chain breaking. The sizes of Na–ClO_4_ clusters, which average ∼2.5 formula units initially, increase significantly on applying pressure from the *z* direction compared to applying pressure from the *x* and *y* directions (Fig. S8b†). Combining the size and numbers of clusters on a histogram (Fig. 9) suggests that high pressure in the *z* direction is responsible for more severe chain breaking and also more expulsion of DMF molecules from the Na^+^ coordination sphere in the cocrystals. The removal of these DMF molecules results in the formation of new Na–ClO_4_ clusters, which is consistent with the observed experimental conversion of the 3 : 1 stoichiometry to 2 : 1 under applied pressure. Similar to the thermal conversion case, the pressure induced complete conversion is difficult to observe computationally on such short timescales based on the limitations of simulation models, but providing visulaization for inferences on the molecular-level details on the process of thermal or pressure-induced juicing.

## 4. Conclusions

Presented here is a description of the behavior of the solvate sponge crystal and an explanation of the reversible stoichiometric conversion/juicing and melting behavior of (DMF)_3_NaClO_4_. XRD indicates the conversion of the 3 : 1 solvate to the 2 : 1 solvate upon heating or compression. Visually, this transformation is accompanied by visible liquid DMF expulsion from the crystal lattice. Upon the release of pressure or re-cooling of a heated mixture, the DMF is reabsorbed and the 3 : 1 phase is regenerated. The facility of press casting this material into solid pellets is attributed to this unique materials property. Using MD simulations, the molecular level description of the size and number of ion–ion and ion–solvent clusters describe the atomistic pathway toward this conversion, although we did not observe a direct transformation of 3 : 1 to 2 : 1 stoichiometry at simulated timescales. An atomic scale model of crystal melting, the role of interionic/ion–solvent interactions in stoichiometric conversion, and modeling of pressure induced conversion in this work provides valuable understanding for the development of melt- and press-castable electrolyte materials. Future work will exploit these simulation protocols to examine the molecular mechanism of ion conduction in these press- and melt-castable ion-conducting pellets.

## Author contributions

Conceptualization: MJZ, SLW, AV; formal analysis: PP, BF, SA; funding acquisition: MJZ, SLW, AV, PP; investigation: PP, BF, SA; methodology: BF, PP; project administration: MJZ, SLW, AV; supervision: MJZ, SLW, AV; visualization: MJZ, PP; writing – original draft: PP; writing – review & editing: all authors.

## Conflicts of interest

There are no conflicts to declare.

## Supplementary Material

SC-012-D0SC06455F-s001

SC-012-D0SC06455F-s002

SC-012-D0SC06455F-s003

SC-012-D0SC06455F-s004

SC-012-D0SC06455F-s005
