# Supplementary material for: Solvate sponge crystals of (DMF)3NaClO4: reversible pressure/temperature controlled juicing in a melt/press-castable sodium-ion conductor
Source: Chem Sci. 2021 Mar 1;12(15):5574–81. doi: 10.1039/d0sc06455f (PMC8179650; doi:10.1039/d0sc06455f)
Supplement: SC-012-D0SC06455F-s001 [file SC-012-D0SC06455F-s001.pdf]

Electronic Supplementary Material (ESI) for Chemical Science.
This journal is © The Royal Society of Chemistry 2021

Supporting Information

Solvate Sponge Crystals of $(DMF)_3NaClO_4$: reversible pressure/temperature controlled juicing in a melt/press-castable sodium-ion conductor.

Prabhat Prakash,[a,c] Ardhra Shylendran,[a] Birane Fall,[b] Michael J. Zdilla,[b] Stephanie L. Wunder,[b]* Arun Venkatnathan.[a]**

[a] Department of Chemistry and Centre for Energy Science, Indian Institute of Science Education and Research, Dr. Homi Bhabha Road, Pashan, Pune: 411008, India.

[b] Department of Chemistry, Temple University, 1901 N. 13th St., Philadelphia, PA 19086, USA.

[c] Materials Science and Engineering, Indian Institute of Technology Gandhinagar, Gujarat 382355, India.

Email:

* Arun Venkatnathan: arun@iiserpune.ac.in
* Michael J. Zdilla: mzdilla@temple.edu
* Stephanie L. Wunder: slwunder@temple.edu

Table of Contents

Standard Operating Procedure (SOP) for potentially explosive hot mixtures. . S3

Chemical and physical properties of 3:1 *vs.* 2:1 stoichiometric cocrystals of DMF-$NaClO_4$ S3

Powder XRD a pressed pellet of DMF-$NaClO_4$ S4

Force-field parameters S6

Details of topology file S7

Mass density and non-bonded interaction energy for model P during simulated heating S8

Snapshots from simulations S9

Cluster analysis for $(DMF)_3NaClO_4$ simulated as model *P*: Histograms at constant T S10

Radial Distribution Functions calculated from simulations on model *P* S11

Differential Scanning Calorimetry of $(DMF)_2NaClO_4$ S12

Effect of high-pressure anisotropicity on the average size of various clusters in cocrystals S13

Effect of high-pressure anisotropicity on the total number of various clusters in cocrystals S14

X-ray Crystallographic Tables for $(DMF)_2NaClO_4$ S15

References S19

Standard Operating Procedure (SOP) for potentially explosive hot mixtures.

Caution: Perchlorate-containing materials are hazardous and can cause explosions, especially at high temperature, and when mixed with organic fuels. While no explosions occurred during our work, the use of explosion proof masks, Kevlar gloves, and an explosion-proof blast shield within a fume hood are recommended when heating perchlorate-organic mixtures. In this work, sodium perchlorate was weighed and combined with liquid DMF using standard PPE (gloves, lab coat, safety glasses), and combined in a Teflon-capped heavy-wall pressure flask. The flask was placed into a temperature-controlled oil bath in a hood, and heated with the hood sash down. We also use and recommend the employment of an additional plexiglass blast shield between the reaction and the hood sash. Once the reaction was complete, the heat was turned off by reaching around the plexiglass shield wearing a pair of Kevlar gloves to turn off the heat. Alternatively, the hot plate may be unplugged from the wall to cease heating. Once the mixture was cooled to room temperature, it was removed from the hood and handled once again with standard PPE.

Chemical and physical properties of 3:1 *vs.* 2:1 stoichiometric cocrystals of DMF-$NaClO_4$

Table S1. Comparison of structural features and melting/decomposition in stoichiometric cocrystals of DMF and $NaClO_4$.

Stoichiometry	3:1[a]	2:1[b]
Crystal System	Hexagonal	Monoclinic
Space Group	P-62c	P2/c
Na---Na distance in primary channel (in Å)	3.23	3.40
Na---Na distance in secondary channel (in Å)	12.00	8.54
Na---O(DMF) distances (in Å)	2.40	2.34, 2.44
Na---ClO_4 distances (in Å)	7.11 (Na---Cl)	2.36, 2.51 (Na---O)
T_m (from DSC) (in ^{0}C)	55	70
T_d (from TGA) (in ^{0}C)	50	40

a. All data is reproduced from Zdilla and coworkers[1].

b. All data, except T_m and T_d, is reproduced from Rao and coworkers[2].

c. DSC data for 2:1 is provided in the Figure S7.

Powder XRD a pressed pellet of DMF-$NaClO_4$

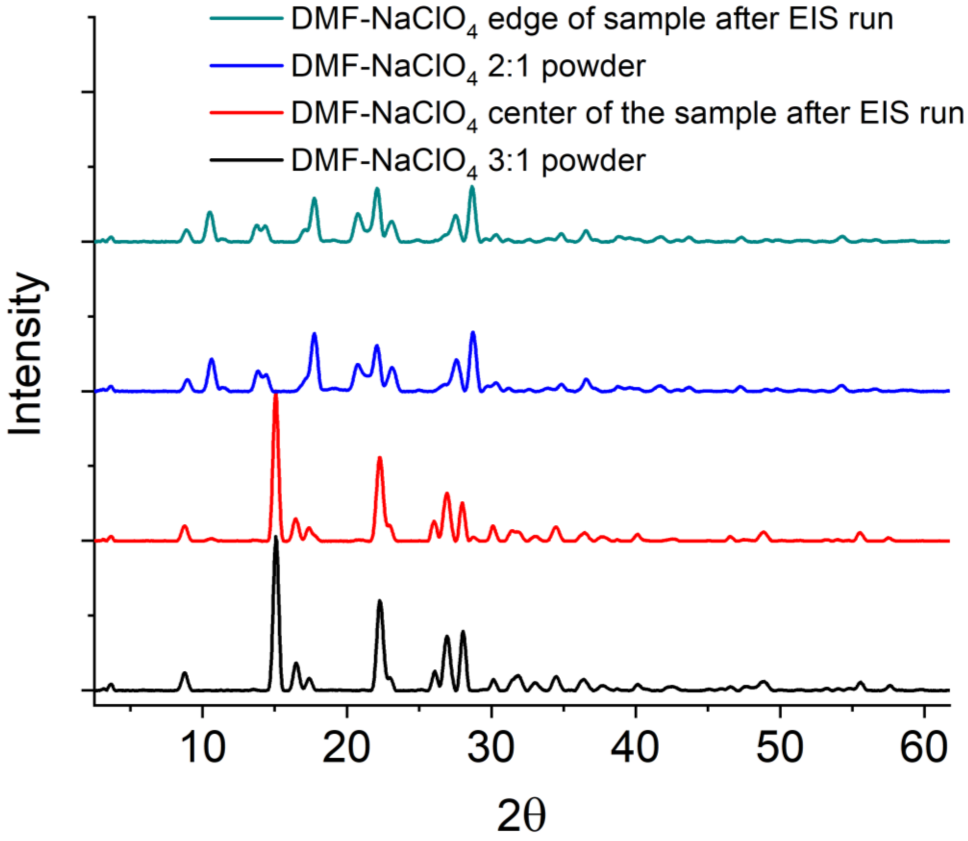

Figure S1. PXRD of $(DMF)_3NaClO_4$ and $(DMF)_2NaClO_4$ samples. Black: Crystalline $(DMF)_3NaClO_4$ before pressing. Red: Following pressing, a sample taken from near the center of the pressed pellet in an EIS cell shows presence of $(DMF)_3NaClO_4$. Blue: experimentally isolated sample of $(DMF)_2NaClO_4$ contaminated by $(DMF)_3NaClO_4$. Green: Sample taken from near the edge (green) of a pressed pellet in an EIS cell shows appearance of $(DMF)_2NaClO_4$.

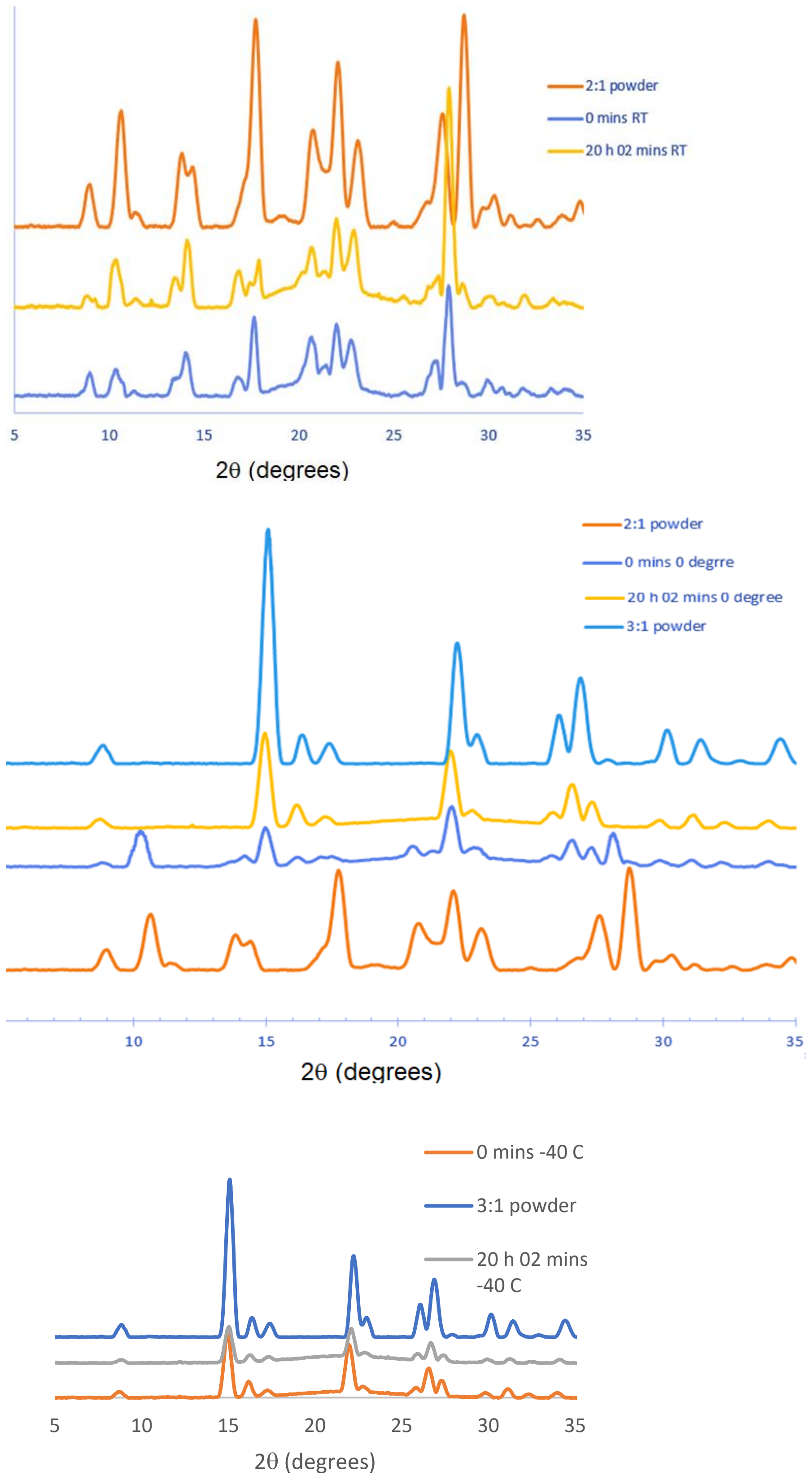

Figure S2. PXRD patterns of post-melted samples of $(DMF)_3NaClO_4$. At room temperature (top), formation of 2:1 $(DMF)_2NaClO_4$ phase is observed, which partially reverts to the 3:1 phase over the course of 20 h. At 0 °C, the sample fully reverts to the 3:1 phase over 20 h. At -40 °C, the structure immediately reverts to the 3:1 phase and remains so.

Force-field parameters

Bonded parameters: From OPLS-AA force field[3]

vdW parameters in the form of Lennard Jones potential: OPLS-AA force field

Electrostatic charges for Coulombic potential-

All gas-phase calculations were performed using Gaussian 16 Rev A.03 software code[4].

Charge on Na^+ ion: Calculated from optimized structure of $[Na(DMF)_6]^+$ in gas phase using MP2//aug-cc-PVDZ method. $q_{Na}{}^+ = + 0.91525\ e^-$

Charge on Cl and O atoms in $ClO4^-$ anion: Calculated from optimized structure of $ClO4^-$ in gas phase using MP2//aug-cc-PVDZ, and scaled by $q_{Na}{}^+$.

$q_{O(ClO4^-)} = -\ 0.4927\ e^-$, $q_{Cl(ClO4^-)} = +\ 1.05555\ e^-$

Charge on atoms in DMF molecule: adapted from Vasudevan et al.[5]

Details of the topology file used for simulations are provided below.

Details of topology file

```
; All charges from CHELPG
; Na charges from NA(DMF)6 structure MP2/aug-cc-PVDZ
; CLO charges from gas phase scaled from NA(DMF)6 str opt MP2//aug-cc-PVDZ
; DMF charges from J. Mol. Liq 206 (2015) 338-342
#include "~/oplsaa.ff/forcefield.itp"
[ moleculetype ]
; Name          nrexcl
NA          1
[ atoms ]
;   nr     type  resnr residue  atom   cgnr    charge      mass
    1   opls_407     1    NA    NA      1      0.91525    22.98977
[ moleculetype ]
; Name          nrexcl
CLO          3
[ atoms ]
;   nr     type  resnr residue  atom   cgnr    charge      mass
    1   opls_998     1    CLO    CL      2      1.05555   35.453
    2   opls_999     1    CLO    O       2     -0.4927   15.9994
    3   opls_999     1    CLO    O       2     -0.4927   15.9994
    4   opls_999     1    CLO    O       2     -0.4927   15.9994
    5   opls_999     1    CLO    O       2     -0.4927   15.9994
[ moleculetype ]
; Name          nrexcl
DMF          3
[ atoms ]
;   nr     type  resnr residue  atom   cgnr    charge      mass
      1  opls_236   1    DMF   O1   1   -0.68000    15.9994
      2  opls_239   1    DMF   N1   1   0.040000    14.0067
      3  opls_235   1    DMF   C1   1   0.500000    12.011
      4  opls_140   1    DMF   H1   1   0.000000    1.008
      5  opls_243   1    DMF   C2   2   -0.11000    12.011
      6  opls_140   1    DMF   H2   2   0.060000    1.008
      7  opls_140   1    DMF   H3   2   0.060000    1.008
      8  opls_140   1    DMF   H4   2   0.060000    1.008
      9  opls_243   1    DMF   C3   3   -0.11000    12.011
     10  opls_140   1    DMF   H5   3   0.060000    1.008
     11  opls_140   1    DMF   H6   3   0.060000    1.008
     12  opls_140   1    DMF   H7   3   0.060000    1.008
```

Mass density and non-bonded interaction energy for model P during simulated heating

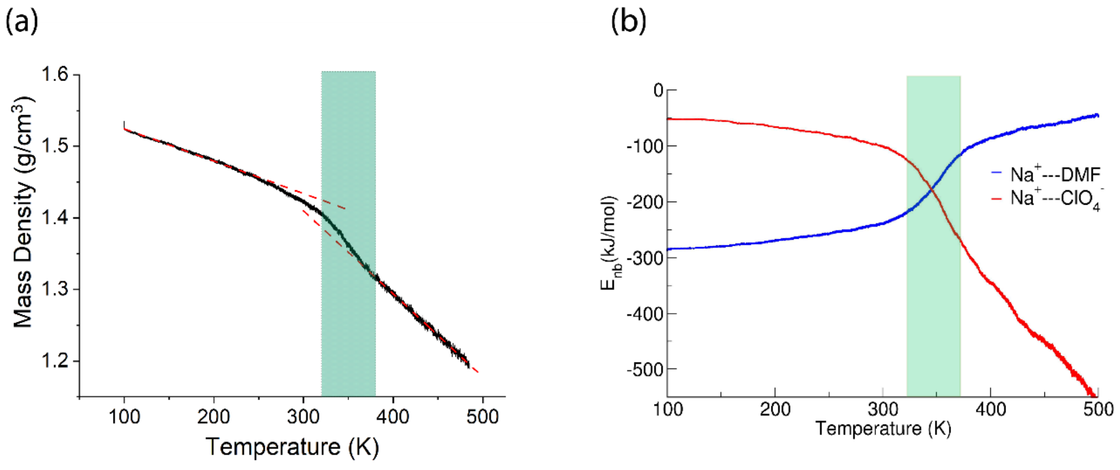

Figure S3. (a) Mass density and **(b)** non-bonded interaction energy E_{nb} of $(DMF)_3NaClO_4$ in model P during simulated heating from 100 K to 500 K with a heating rate of 20 K/ns. The highlighted region shows a rapid drop of density in **a** and extreme change in ion-solvent vs. interionic interactions in **b** during the melting of cocrystals.

Snapshots from simulations

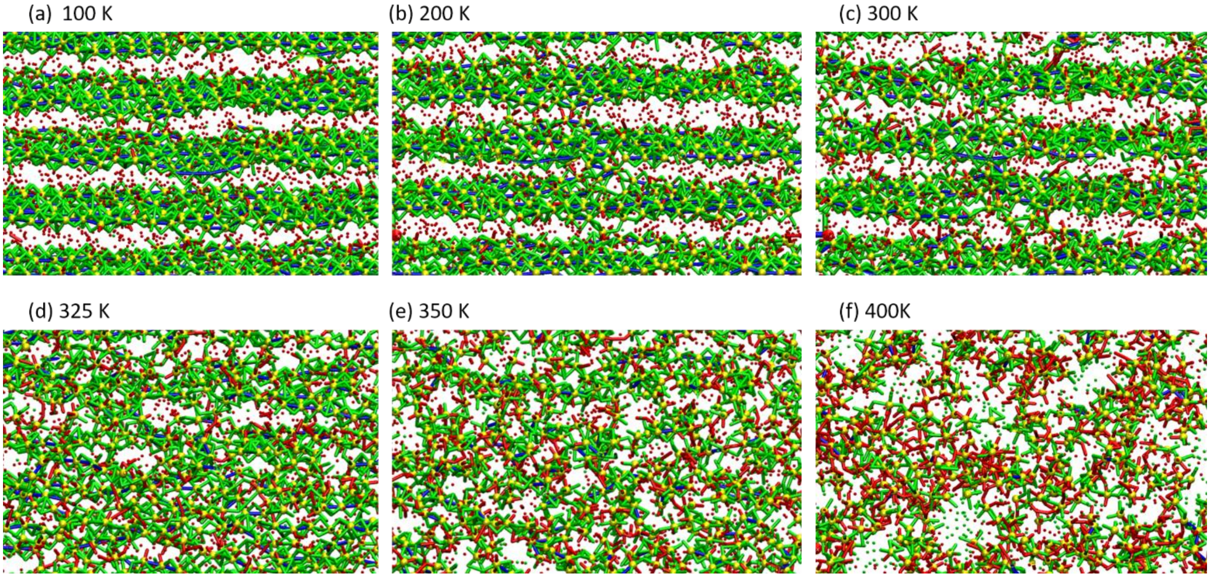

Figure S4. Snapshots of $(DMF)_3NaClO_4$ simulated as model P. Atoms: Yellow- Na^+, Green- O(DMF), Red- O(ClO_4^-); Bonds: Blue- Na...Na, Green- Na...O(DMF), Red- Na...O(ClO_4^-); Cut-off for dynamic bonds: Na…Na ≤ 3.5 Å, Na…O(DMF) ≤ 3.0 Å, Na…O(ClO_4^-) ≤ 2.2 Å. All the snapshots of the trajectory are provided in the Supporting Movie 1.

Cluster analysis for $(DMF)_3NaClO_4$ simulated as model *P*: Histograms at constant T

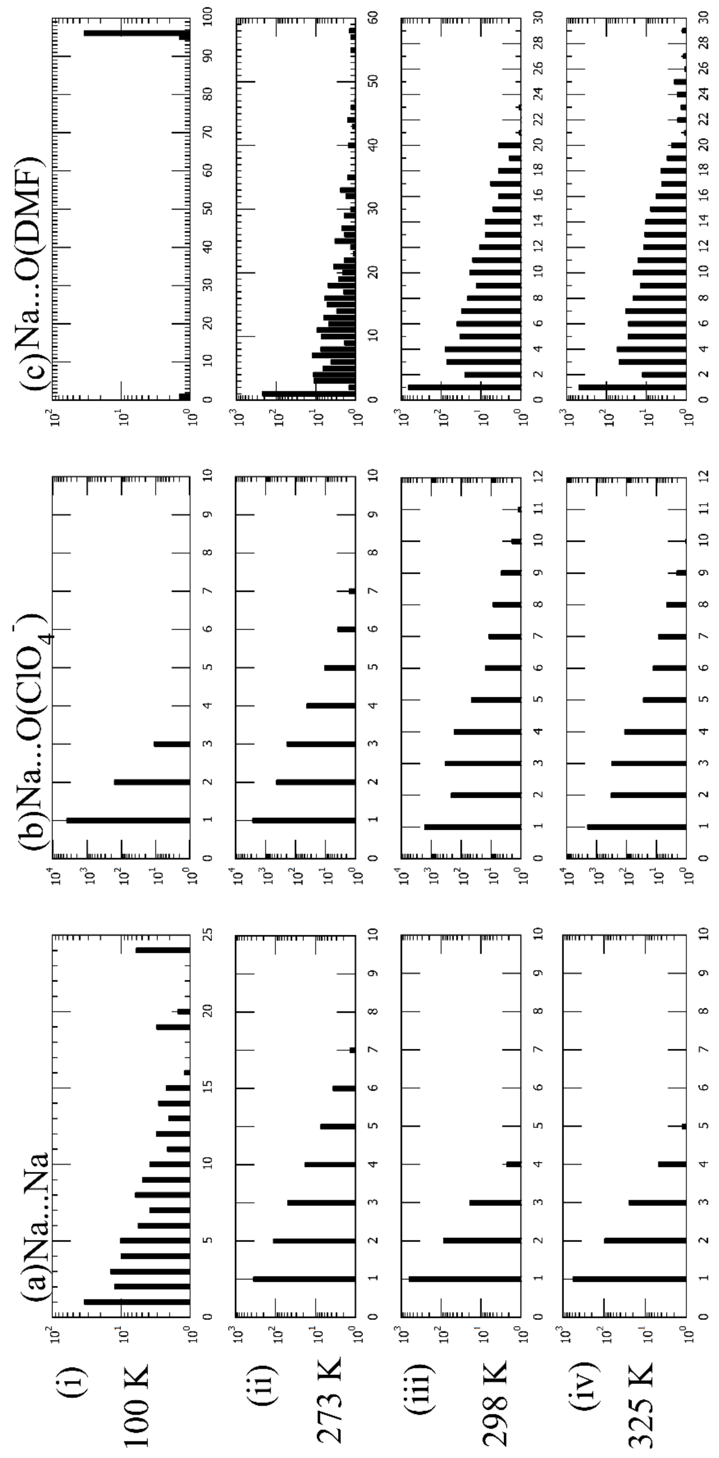

Figure S5. Cluster histograms for $(DMF)_3NaClO_4$ simulated at constant temperature under NpT ensemble conditions (a) Na...Na clusters (≤ 3.5 Å), (b) Na...ClO_4^- clusters (≤ 2.2 Å), (c) Na...DMF clusters (≤ 3.0 Å); (i) 100 K, (ii) 273 K, (iii) 298 K and (iv) 325 K.

Y-axis: number of clusters, X-axis: size of clusters.

Radial Distribution Functions calculated from simulations on model *P*

(a)

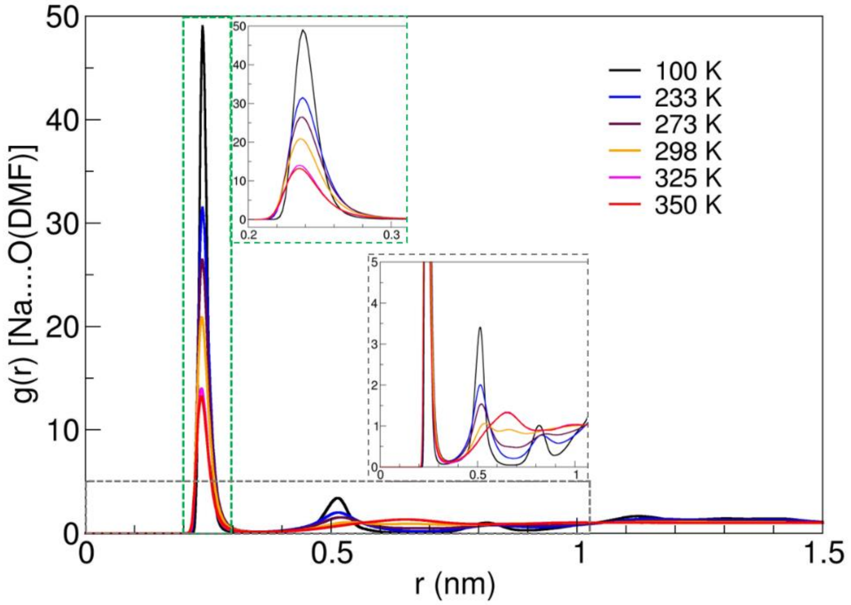

(b)

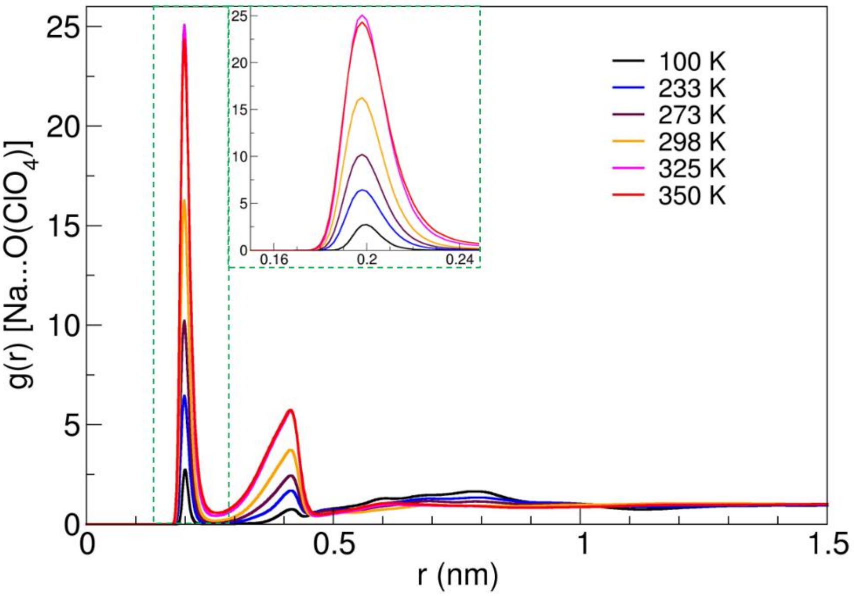

Figure S6. RDF of (a) Na---O(DMF) and (b) Na---O(ClO_4^-) from NPT simulations on model P at various temperatures.

Differential Scanning Calorimetry of $(DMF)_2NaClO_4$

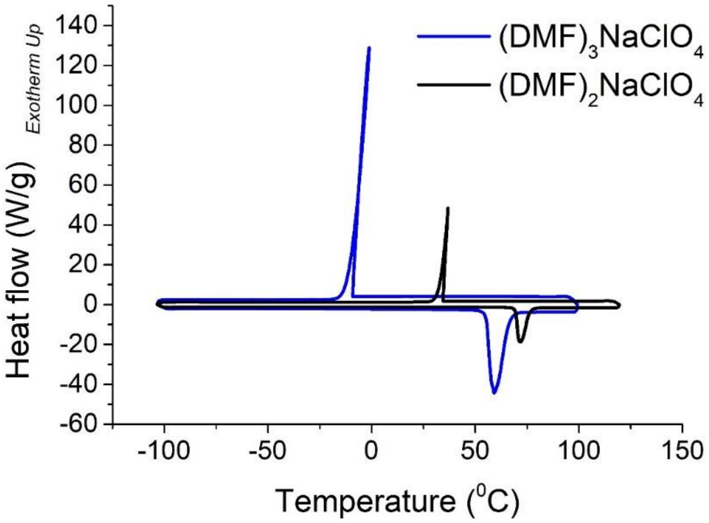

Figure S7. DSC of $(DMF)_2NaClO_4$ at scan rate of 10 ^{0}C/min. Data for $(DMF)_3NaClO_4$ is reused for comparison from Zdilla and coworkers[1] © 2016 Wiley-VCH Verlag GmbH &Co. KGaA,Weinheim.

Effect of high-pressure anisotropicity on the average size of various clusters in cocrystals

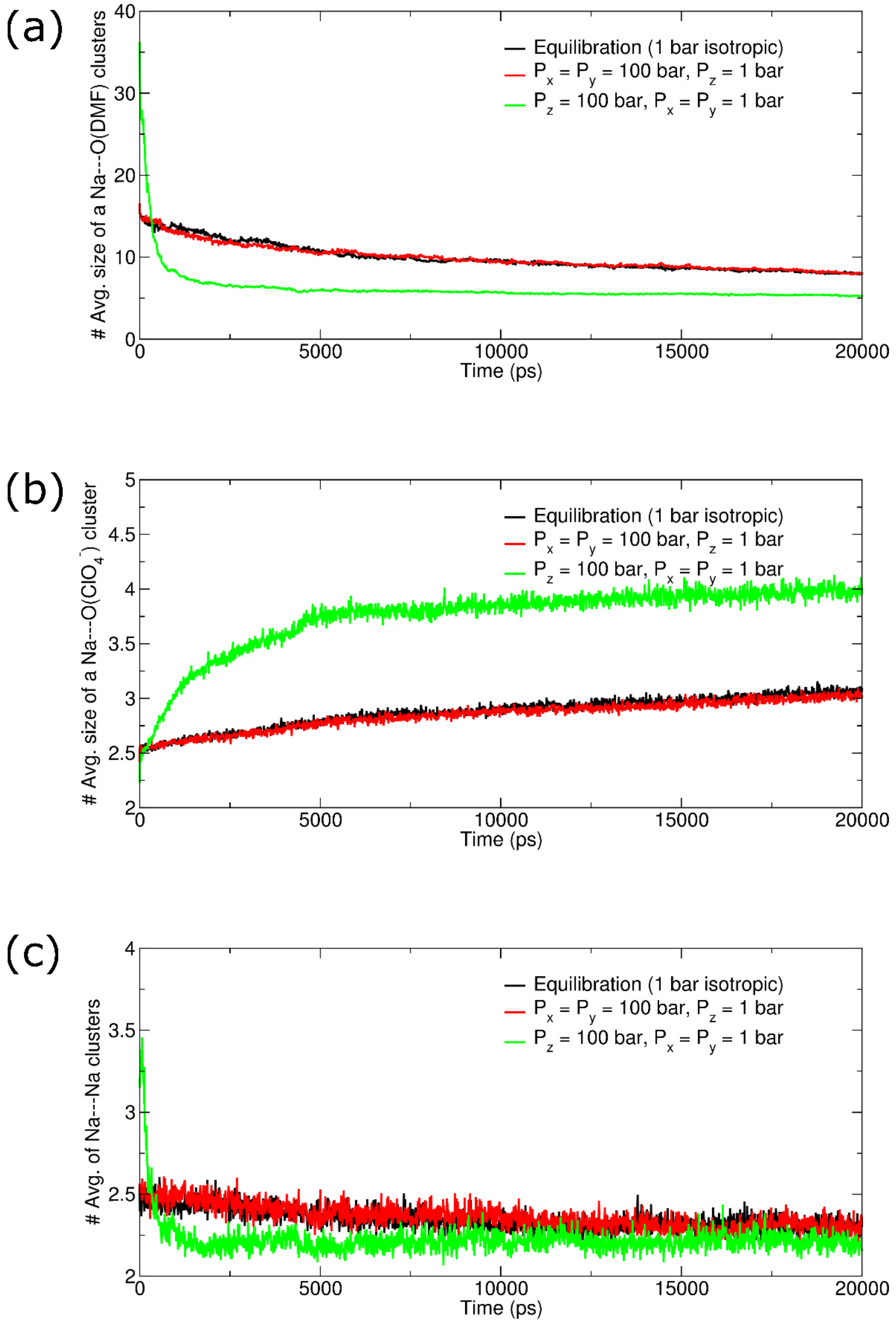

Figure S8. Average size of **(a)** Na---O(DMF) (≤ 3.0 Å), **(b)** Na---O(ClO_4^-) (≤ 2.2 Å), and **(c)** Na---Na (≤ 3.5 Å), for a 20 ns trajectory for isotropic 1 bar equilibration compared with 100 bar anisotropic pressure from xy and z directions, at T = 298 K.

Effect of high-pressure anisotropicity on the total number of various clusters in cocrystals

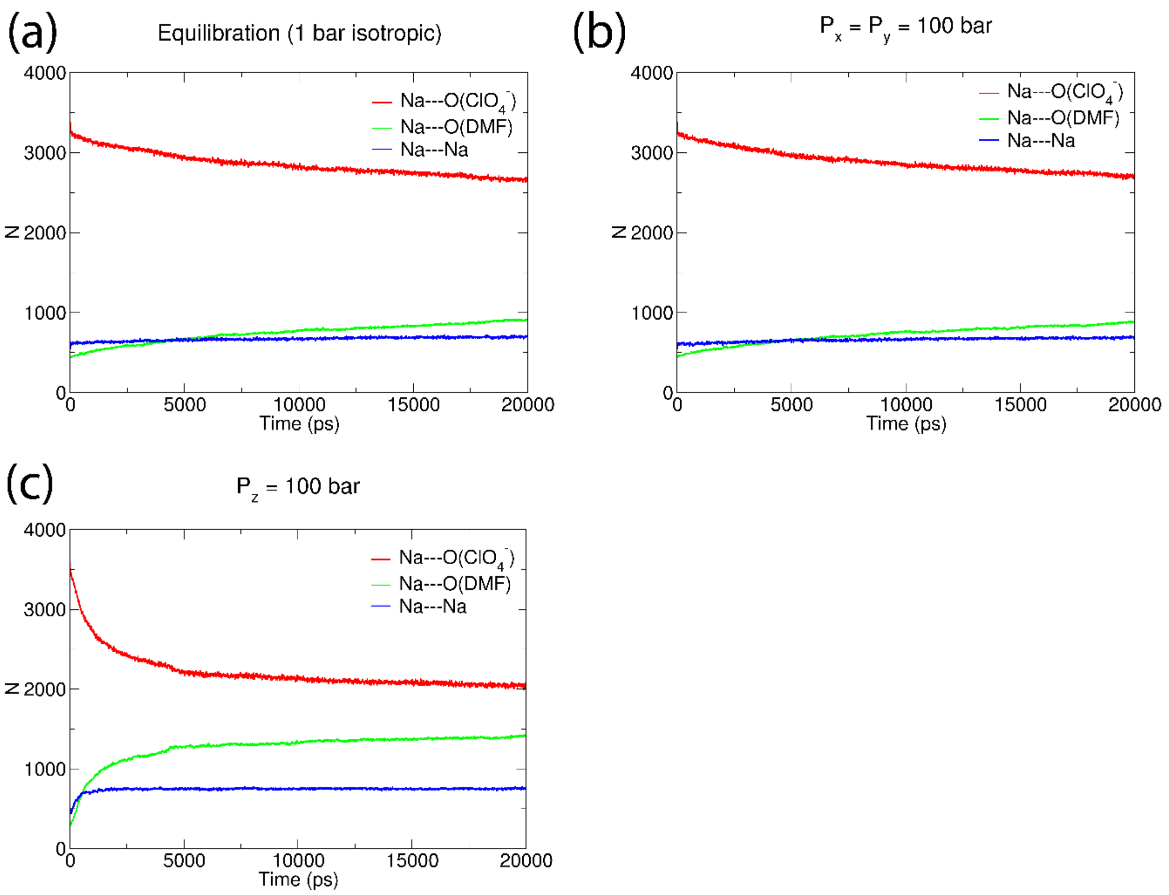

Figure S9. Number of Na---O(DMF) (≤ 3.0 Å), Na---O(ClO_4^-) (≤ 2.2 Å), and Na---Na (≤ 3.5 Å), for a 20 ns trajectory simulated under *NpT* conditions as **(a)** P = 1 bar isotropic, **(b)** P_x = P_y = 100 bar, P_z = 1 bar, and **(c)** P_x = P_y = 1 bar, P_z = 100 bar, at T = 298 K.

X-ray Crystallographic Tables for $(DMF)_2NaClO_4$

Table S2 Crystal data and structure refinement for mo_3183_0m.

Empirical formula	$C_6H_{14}N_2O_6NaCl$
Formula weight	268.63
Temperature/K	99.98
Crystal system	monoclinic
Space group	P2/n
a/Å	9.1493(14)
b/Å	10.1833(15)
c/Å	13.220(2)
α/°	90
β/°	108.244(3)
γ/°	90
Volume/Å^3	1169.8(3)
Z	4
ρ_{calc}g/cm^3	1.525
μ/mm^{-1}	0.378
F(000)	560.0
Crystal size/mm^3	0.152 × 0.121 × 0.076
Radiation	MoKα (λ = 0.71073)
2Θ range for data collection/°	4 to 55.62
Index ranges	$-5 \leq h \leq 11$, $-10 \leq k \leq 13$, $-17 \leq l \leq 16$
Reflections collected	5501
Independent reflections	2335 [R_{int} = 0.0157, R_{sigma} = 0.0210]
Data/restraints/parameters	2335/0/159
Goodness-of-fit on F^2	1.059
Final R indexes [I>=2σ (I)]	R_1 = 0.0320, wR_2 = 0.0816
Final R indexes [all data]	R_1 = 0.0414, wR_2 = 0.0869
Largest diff. peak/hole / e Å^{-3}	0.50/-0.30

Table S3 Bond Lengths for mo_3183_0m.

Atom	Atom	Length/Å	Atom	Atom	Length/Å
Cl1	O1C	1.4391(14)	Na2	O1[2]	2.3390(13)
Cl1	O2C	1.4227(17)	Na2	O2	2.4416(13)
Cl1	O3C	1.4315(16)	Na2	O2[2]	2.4416(13)
Cl1	O4C	1.4375(12)	Na2	O4C[2]	2.5094(15)
Na1	Na2[1]	3.4822(5)	Na2	O4C	2.5094(15)
Na1	Na2	3.4822(5)	O1	C1	1.239(2)
Na1	O1[1]	2.3782(12)	O2	C2	1.236(2)
Na1	O1	2.3782(12)	N1	C1	1.323(3)
Na1	O1C[1]	2.3572(16)	N1	C1T	1.460(2)
Na1	O1C	2.3572(16)	N1	C2T	1.458(2)
Na1	O2[1]	2.3398(12)	N2	C2	1.322(2)
Na1	O2	2.3398(12)	N2	C3T	1.460(3)
Na2	O1	2.3390(13)	N2	C4T	1.459(2)

[1]1-X,1-Y,-Z; [2]3/2-X,+Y,1/2-Z

Table S4 Bond Angles for mo_3183_0m.

Atom	Atom	Atom	Angle/°	Atom	Atom	Atom	Angle/°
O2C	Cl1	O1C	109.20(12)	O1	Na2	O1[2]	164.50(7)
O2C	Cl1	O3C	109.67(10)	O1	Na2	O2[2]	111.13(5)
O2C	Cl1	O4C	110.26(10)	O1[2]	Na2	O2[2]	80.84(4)
O3C	Cl1	O1C	108.90(10)	O1[2]	Na2	O2	111.13(5)
O3C	Cl1	O4C	110.20(9)	O1	Na2	O2	80.84(4)
O4C	Cl1	O1C	108.59(8)	O1	Na2	O4C[2]	81.65(5)
Na2[1]	Na1	Na2	180.00(3)	O1[2]	Na2	O4C[2]	87.06(5)
O1	Na1	Na2[1]	138.01(3)	O1[2]	Na2	O4C	81.65(5)
O1[1]	Na1	Na2	138.01(3)	O1	Na2	O4C	87.06(5)
O1	Na1	Na2	41.99(3)	O2	Na2	Na1[2]	112.46(4)
O1[1]	Na1	Na2[1]	41.99(3)	O2[2]	Na2	Na1	112.46(4)

O1[1]	Na1	O1	180.0	O2[2]	Na2	Na1[2]	42.12(3)
O1C[1]	Na1	Na2	107.99(4)	O2	Na2	Na1	42.12(3)
O1C	Na1	Na2	72.01(4)	O2	Na2	O2[2]	83.43(6)
O1C	Na1	Na2[1]	107.99(4)	O2[2]	Na2	O4C[2]	97.95(4)
O1C[1]	Na1	Na2[1]	72.01(4)	O2	Na2	O4C	97.95(4)
O1C[1]	Na1	O1[1]	88.58(5)	O2[2]	Na2	O4C	161.68(5)
O1C[1]	Na1	O1	91.42(5)	O2	Na2	O4C[2]	161.68(5)
O1C	Na1	O1	88.58(5)	O4C[2]	Na2	Na1	122.70(3)
O1C	Na1	O1[1]	91.42(5)	O4C	Na2	Na1	79.09(3)
O1C	Na1	O1C[1]	180.00(8)	O4C[2]	Na2	Na1[2]	79.09(3)
O2[1]	Na1	Na2	135.58(3)	O4C	Na2	Na1[2]	122.70(3)
O2	Na1	Na2[1]	135.58(3)	O4C[2]	Na2	O4C	86.47(8)
O2[1]	Na1	Na2[1]	44.42(3)	Na2	O1	Na1	95.15(5)
O2	Na1	Na2	44.42(3)	C1	O1	Na1	120.71(10)
O2	Na1	O1	82.17(4)	C1	O1	Na2	123.41(11)
O2[1]	Na1	O1	97.83(4)	Cl1	O1C	Na1	137.31(11)
O2	Na1	O1[1]	97.83(4)	Na1	O2	Na2	93.46(4)
O2[1]	Na1	O1[1]	82.17(4)	C2	O2	Na1	126.38(11)
O2[1]	Na1	O1C[1]	86.44(6)	C2	O2	Na2	133.15(10)
O2[1]	Na1	O1C	93.56(6)	Cl1	O4C	Na2	127.37(8)
O2	Na1	O1C[1]	93.56(6)	C1	N1	C1T	121.13(16)
O2	Na1	O1C	86.44(6)	C1	N1	C2T	121.70(15)
O2	Na1	O2[1]	180.0	C2T	N1	C1T	117.15(17)
Na1[2]	Na2	Na1	152.12(3)	C2	N2	C3T	121.52(15)
O1[2]	Na2	Na1	142.94(3)	C2	N2	C4T	121.4(2)
O1	Na2	Na1[2]	142.94(3)	C4T	N2	C3T	117.02(19)
O1[2]	Na2	Na1[2]	42.86(3)	O1	C1	N1	124.87(16)
O1	Na2	Na1	42.86(3)	O2	C2	N2	124.83(17)

[1]1-X,1-Y,-Z; [2]3/2-X,+Y,1/2-Z

Experimental

Single crystals of $C_6H_{14}N_2O_6NaCl$ were mounted on a Bruker APEX-II CCD diffractometer. The crystal was kept at 99.98 K during data collection. The structure was solved with the ShelXS[7] structure solution program using Direct Methods and refined with the ShelXL[8] refinement package using Least Squares minimization using Olex2[6]as a GUI.

Crystal structure determination of $(DMF)_2NaClO_4$

Crystal Data for $C_6H_{14}N_2O_6NaCl$ (M =268.63 g/mol): monoclinic, space group P2/n (no. 13), a = 9.1493(14) Å, b = 10.1833(15) Å, c = 13.220(2) Å, β = 108.244(3)°, V = 1169.8(3) Å^3, Z = 4, T = 99.98 K, μ(MoKα) = 0.378 mm^{-1}, $Dcalc$ = 1.525 g/cm^3, 5501 reflections measured (4° ≤ 2Θ ≤ 55.62°), 2335 unique (R_{int} = 0.0157, R_{sigma} = 0.0210) which were used in all calculations. The final R_1 was 0.0320 (I > 2σ(I)) and wR_2 was 0.0869 (all data).

Refinement model description

Number of restraints - 0, number of constraints - unknown.

Details:

```
1. Fixed Uiso
 At 1.5 times of:
  All C(H,H,H) groups
2.a Idealised Me refined as rotating group:
 C1T(H1TA,H1TB,H1TC), C2T(H2TA,H2TB,H2TC), C3T(H3TA,H3TB,H3TC), C4T(H4TA,H4TB,
 H4TC)
```

References

1 P. R. Chinnam, B. Fall, D. A. Dikin, A. Jalil, C. R. Hamilton, S. L. Wunder and M. J. Zdilla, *Angew. Chemie Int. Ed.*, 2016, **55**, 15254–15257.

2 C. Pulla Rao, A. Muralikrishna Rao and C. N. R. Rao, *Inorg. Chem.*, 1984, **23**, 2080–2085.

3 W. L. Jorgensen, D. S. Maxwell and J. Tirado-Rives, *J. Am. Chem. Soc.*, 1996, **118**, 11225–11236.

4 M. J. Frisch, G. W. Trucks, H. B. Schlegel, G. E. Scuseria, M. A. Robb, J. R. Cheeseman, G. Scalmani, V. Barone, G. A. Petersson, H. Nakatsuji, X. Li, M. Caricato, A. V Marenich, J. Bloino, B. G. Janesko, R. Gomperts, B. Mennucci, H. P. Hratchian, J. V Ortiz, A. F. Izmaylov, J. L. Sonnenberg, Williams, F. Ding, F. Lipparini, F. Egidi, J. Goings, B. Peng, A. Petrone, T. Henderson, D. Ranasinghe, V. G. Zakrzewski, J. Gao, N. Rega, G. Zheng, W. Liang, M. Hada, M. Ehara, K. Toyota, R. Fukuda, J. Hasegawa, M. Ishida, T. Nakajima, Y. Honda, O. Kitao, H. Nakai, T. Vreven, K. Throssell, J. A. Montgomery Jr., J. E. Peralta, F. Ogliaro, M. J. Bearpark, J. J. Heyd, E. N. Brothers, K. N. Kudin, V. N. Staroverov, T. A. Keith, R. Kobayashi, J. Normand, K. Raghavachari, A. P. Rendell, J. C. Burant, S. S. Iyengar, J. Tomasi, M. Cossi, J. M. Millam, M. Klene, C. Adamo, R. Cammi, J. W. Ochterski, R. L. Martin, K. Morokuma, O. Farkas, J. B. Foresman and D. J. Fox, 2016, Gaussian 16 Rev. A.03.

5 V. Vasudevan and S. H. Mushrif, *J. Mol. Liq.*, 2015, **206**, 338–342.

6 O.V. Dolomanov, L.J. Bourhis, R.J, Gildea, J.A.K Howard, H. Puschmann, *J. Appl. Cryst.* 2009, **42**, 339-341.

7 G. M. Sheldrick, *Acta Cryst*., 2008 A64, 112-122.

8 G. M. Sheldrick, *Acta Cryst*., 2015 C71, 3-8.
